# Comparing the Efficacy of Intravenous Versus Oral Iron Supplementation for Anemic Patients With Inflammatory Bowel Disease: A Meta-Analysis

**DOI:** 10.7759/cureus.97917

**Published:** 2025-11-27

**Authors:** Basim S Alharbi, Bashair Z Alqaidi, Fouad S Alharbi, Ahmed M Alharbi, Mohammed S Alsharif

**Affiliations:** 1 Gastroenterology, Imam Abdulrahman Al Faisal Hospital, Riyadh, SAU; 2 Clinical Pharmacy, King Salman Bin Abdulaziz Medical City, Medina, SAU; 3 Pharmacy, Prince Mohammad Bin Abdulaziz Hospital, Medina, SAU; 4 Pharmacy, Prince Sultan Armed Forces Hospital, Medina, SAU; 5 Nursing, Prince Mohammad Bin Abdulaziz Hospital, Medina, SAU

**Keywords:** inflammatory bowel disease, intravenous iron, iron-refractory iron deficiency anemia, meta-analysis, oral iron

## Abstract

Iron deficiency anemia (IDA) is a frequent complication in inflammatory bowel diseases (IBDs) and adversely affects patient outcomes and quality of life. Oral iron supplementation is widely used but can be poorly tolerated and less effective during active inflammation. Intravenous (IV) iron supplementation bypasses intestinal absorption barriers, but its comparative efficacy in IBD remains under debate.

This study aims to compare the efficacy of IV versus oral iron supplementation in achieving effective hemoglobin increases in patients with IBD-associated anemia. A systematic search of PubMed, Web of Science, Scopus, Medline, the Cochrane Library, and Google Scholar was conducted up to March 2022, yielding 711 records. After removing duplicates and applying inclusion criteria, seven studies (six randomized controlled trials and one prospective study), involving 1,029 patients, were included. Data were pooled using a fixed-effects model to calculate odds ratios (ORs) with 95% confidence intervals (CIs). The primary outcome was the proportion of patients achieving a clinically meaningful hemoglobin increase. Heterogeneity was assessed using the I² statistic, and publication bias was evaluated using funnel plots. Across the seven included studies, IV iron supplementation was significantly more effective than oral iron in increasing hemoglobin levels in IBD-associated anemia. The pooled OR was 1.45 (95% CI: 1.11-1.90; p = 0.006), favoring IV iron. Heterogeneity was minimal (I² = 2%, p = 0.41). Funnel plot analysis showed symmetrical distribution, suggesting a low likelihood of publication bias. In conclusion, IV iron supplementation provides a statistically and clinically significant advantage over oral iron for treating anemia in patients with IBD, with consistent results across diverse populations and minimal heterogeneity. These findings support guideline recommendations favoring IV iron, particularly in patients with active disease, intolerance to oral formulations, or the need for rapid anemia correction.

## Introduction and background

Anemia is a common and clinically significant extraintestinal manifestation of inflammatory bowel diseases (IBDs), which include ulcerative colitis and Crohn’s disease [1]. Its prevalence in this population is considerably higher than in the general population, with studies estimating rates between 20% and 70%, depending on disease activity, geographic location, and diagnostic criteria used. Anemia in IBD is multifactorial, arising from chronic intestinal inflammation, impaired iron absorption, occult or overt gastrointestinal blood loss, and nutritional deficiencies [1,2]. Among the causes, iron deficiency anemia (IDA) is the most frequent, often compounded by anemia of chronic disease, driven by inflammatory cytokines such as interleukin-6 that alter iron homeostasis through hepcidin upregulation. Left untreated, anemia contributes to fatigue, cognitive dysfunction, impaired physical performance, reduced quality of life, and poor disease outcomes in IBD patients [2,3].

Iron supplementation is the mainstay of therapy for IDA in IBD. However, the optimal route of administration - intravenous (IV) versus oral - remains an important clinical question [4]. Oral iron supplementation is traditionally used due to its low cost, ease of administration, and widespread availability. It is typically administered in the form of ferrous salts or newer formulations like ferric maltol. While oral iron can correct iron deficiency in some IBD patients, its absorption may be limited by active inflammation, and gastrointestinal side effects such as abdominal discomfort, nausea, constipation, and diarrhea are common. Moreover, unabsorbed luminal iron can exacerbate intestinal inflammation by altering the gut microbiota composition and generating reactive oxygen species, potentially aggravating IBD symptoms [1,3-5].

IV iron supplementation offers an alternative route that bypasses the inflamed gastrointestinal tract, enabling rapid repletion of iron stores. IV formulations - such as ferric carboxymaltose, iron sucrose, and iron isomaltoside - allow the administration of larger doses over shorter periods, often leading to quicker hemoglobin recovery [6]. IV therapy is particularly indicated in cases of severe anemia, intolerance to oral iron, active disease with significant mucosal inflammation, or when rapid correction is clinically necessary. Nevertheless, IV iron is more resource-intensive, requires healthcare facility visits, carries a risk of infusion-related reactions, and is associated with higher costs compared to oral supplementation [4,6].

Clinical guidelines from major gastroenterology societies, such as the European Crohn’s and Colitis Organisation (ECCO) and the British Society of Gastroenterology (BSG), recommend tailoring iron therapy to the severity of anemia, disease activity, and patient tolerance [2]. For example, ECCO suggests that IV iron should be the first-line treatment for patients with severe anemia (hemoglobin < 10 g/dL), active IBD, or intolerance to oral iron, whereas oral iron may be appropriate in patients with mild anemia and quiescent disease. However, despite these recommendations, there remains considerable variability in clinical practice worldwide, partly due to differing healthcare resources, patient preferences, and uncertainties regarding the comparative efficacy and safety of both routes of administration [7,8].

Randomized controlled trials (RCTs) and observational studies have evaluated the effectiveness of oral versus IV iron supplementation in IBD patients, but findings have not been entirely consistent. Some trials have demonstrated superior hemoglobin response rates and faster iron store replenishment with IV iron, while others have found no significant differences in final hematologic outcomes. Furthermore, safety profiles differ, with oral iron being more commonly associated with gastrointestinal side effects, whereas IV iron has a low but notable risk of hypersensitivity reactions and transient hypophosphatemia [3,9-11].

Another layer of complexity arises from heterogeneity in the populations studied, including differences in baseline anemia severity, disease subtype (ulcerative colitis vs. Crohn’s disease), and concomitant therapies, such as biologics or corticosteroids. Variations in the definitions of treatment success, follow-up durations, and iron formulations used also contribute to mixed evidence [10,12,13].

Given the high burden of anemia in IBD and the substantial impact it has on patients’ functional status, optimizing iron therapy is a priority. A robust, disease-specific evidence synthesis comparing IV and oral supplementation is crucial for guiding clinical decision-making and improving patient care. Such an analysis would provide clarity on the relative efficacy, safety, and tolerability of both routes in the IBD context, potentially informing more standardized treatment algorithms and ensuring that patients receive the most appropriate, effective, and tolerable therapy. This study aims to systematically compare the efficacy and safety of IV versus oral iron supplementation in treating anemia among patients with IBD.

## Review

Materials and methods

The methodology followed the Preferred Reporting Items for Systematic Reviews and Meta-Analyses (PRISMA) guidelines to ensure transparency and reproducibility [14]. The research question was framed according to the Population, Intervention, Comparison, and Outcome (PICO) criteria: the population included patients with IBD and IDA, the intervention was IV iron supplementation, the comparison was oral iron supplementation, and the primary outcome was the proportion of patients achieving a clinically meaningful increase in hemoglobin.

Search Strategy

A comprehensive literature search was performed across six electronic databases: PubMed, Web of Science, Scopus, Medline, the Cochrane Library, and Google Scholar. The search encompassed all studies published up to the most recent update in March 2022, without language restrictions at the initial stage. The search strategy combined relevant Medical Subject Headings (MeSH) and free-text terms, including “inflammatory bowel disease,” “Crohn’s disease,” “ulcerative colitis,” “iron deficiency anemia,” “intravenous iron,” “oral iron,” “ferrous sulfate,” “ferric carboxymaltose,” “iron sucrose,” and “randomized controlled trial.” Boolean operators “AND” and “OR” were used to combine terms and ensure optimal retrieval. Reference lists of included articles and relevant reviews were also manually screened to identify additional eligible studies.

Studies were considered eligible if they met the following criteria: (1) enrolled adult or pediatric patients with a confirmed diagnosis of IBD (Crohn’s disease or ulcerative colitis) and anemia, defined by study-specific hemoglobin thresholds; (2) directly compared IV iron supplementation with oral iron supplementation; (3) reported the proportion of patients achieving a clinically meaningful increase in hemoglobin as a dichotomous outcome; and (4) were designed as RCTs, controlled clinical trials, or prospective observational studies. Studies were excluded if they lacked a direct comparison between IV and oral iron, involved patients with anemia of other etiologies without subgroup analysis for IBD, or failed to provide extractable quantitative data for the primary outcome.

Data Extraction

Data were extracted independently by two reviewers using a standardized data collection form. Extracted information included the first author’s name, year of publication, study country or countries, study design, follow-up duration, total sample size, number of participants in the IV and oral groups, mean or range of participant ages in each group, gender distribution, type and dose of iron preparation used, and the proportion of patients achieving an effective hemoglobin increase in each arm. Any discrepancies in extracted data were resolved through discussion and consensus, with the involvement of a third reviewer when necessary. When required data were missing or unclear, attempts were made to contact the study authors for clarification.

Outcome Measures

The primary outcome of interest was the proportion of patients achieving a clinically meaningful increase in hemoglobin level following iron supplementation. While the precise definition of “effective hemoglobin increase” varied slightly between studies, most defined it as either an increase of ≥2 g/dL from baseline or normalization of hemoglobin to within the reference range for sex. For the purposes of pooling data, these definitions were accepted as equivalent, given their clinical relevance and comparability.

Statistical Analysis

Data analysis was conducted using Review Manager (RevMan) version 5.4 (Cochrane Collaboration, Copenhagen, Denmark). For the primary outcome, odds ratios (ORs) with 95% confidence intervals (CIs) were calculated for each study using the Mantel-Haenszel method under a fixed-effects model, as statistical heterogeneity was minimal [15]. Heterogeneity among studies was assessed using the Chi-square test and quantified with the I² statistic, with values of 25%, 50%, and 75% representing low, moderate, and high heterogeneity, respectively. A p-value of <0.10 for the Chi-square test was considered indicative of significant heterogeneity.

The methodological quality of the included RCTs was evaluated using the Cochrane Collaboration’s Risk of Bias tool, which assesses potential bias across seven standard domains: randomization process, allocation concealment, blinding of participants and personnel, blinding of outcome assessment, completeness of outcome data, selective reporting, and other potential sources of bias. Each domain was independently reviewed and categorized as presenting a low risk (“+”), high risk (“-”), or unclear risk (“?”) of bias, based on the information provided in the published articles.

Publication bias was evaluated visually through funnel plot inspection, with symmetry suggesting a low risk of bias. Sensitivity analyses were planned to assess the impact of excluding non-RCTs, varying follow-up durations, and outlier studies on the pooled estimates. Statistical significance was defined as a two-sided p-value of <0.05.

Results

**Figure 1:**
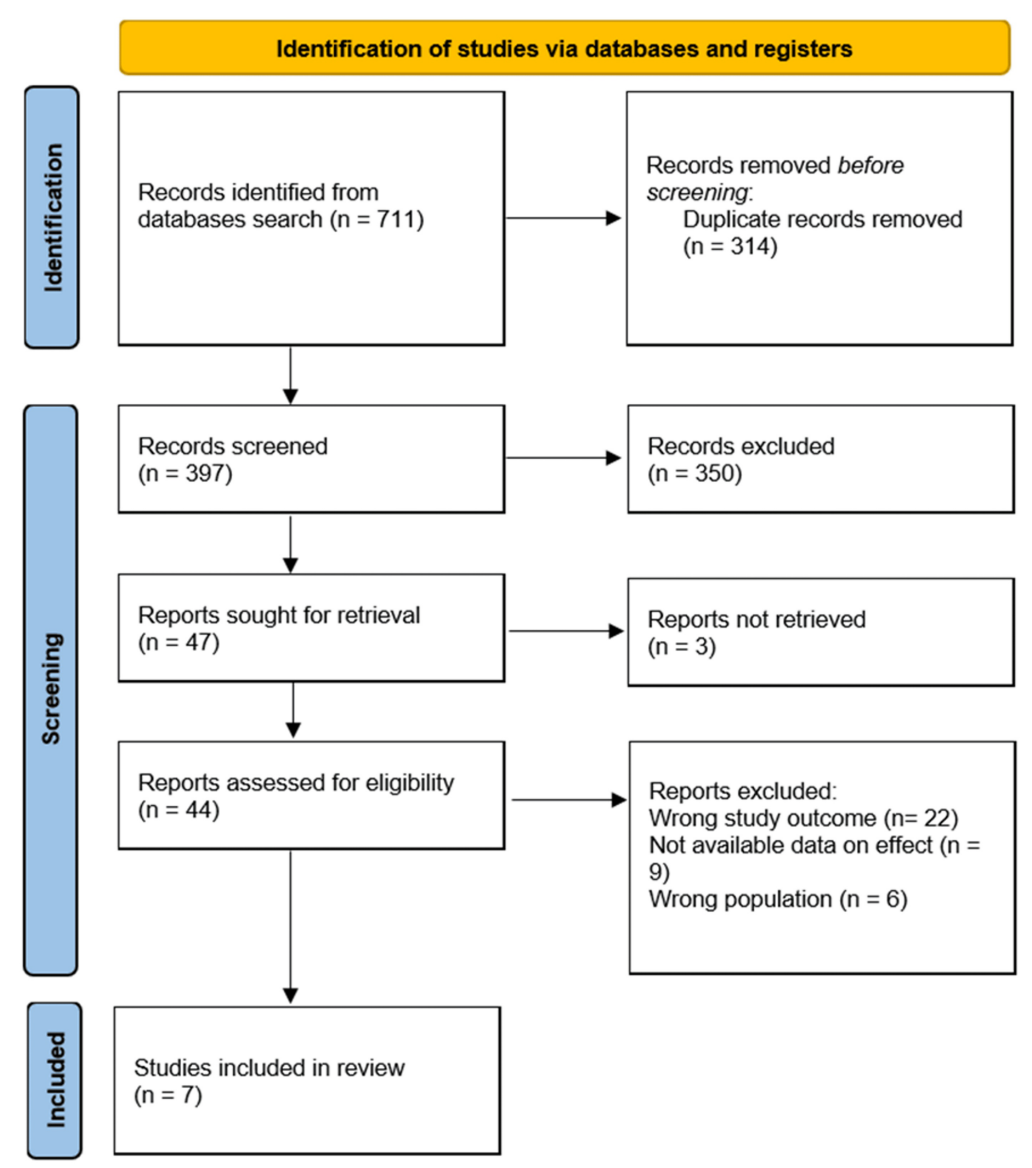
PRISMA flow chart for the summary of the search and screening processes PRISMA: Preferred Reporting Items for Systematic Reviews and Meta-Analyses

The initial search yielded a total of 711 records. After removal of 314 duplicates, 397 titles and abstracts were screened for relevance. Of these, 350 were excluded because they did not meet the inclusion criteria or were clearly unrelated to the research question. Forty-seven studies were retrieved for full-text review, but three could not be accessed despite attempts to contact the authors. The remaining 44 full-text articles were assessed for eligibility, leading to the exclusion of 37 studies due to reasons including inappropriate study design, lack of direct comparison, absence of relevant outcome data, or inclusion of mixed patient populations without separate reporting for IBD. Ultimately, seven studies met all inclusion criteria and were included in the meta-analysis. The study selection process is illustrated in the PRISMA flow diagram (Figure 1).

Characteristics and Findings of the Included Studies

Table 1 provides a summary of the included studies.

**Table 1:** Characteristics of the included studies (n = 7) RCT: Randomized Controlled Trial; IV: Intravenous Iron Supplementation Group; Oral/PO: Oral Iron Supplementation Group; NA: Not Applicable

Study	Country	Design	Follow up (weeks)	Total participants	IV total	Oral total	Age IV (years)	Age oral (years)	Males IV (%)	Males oral (%)	Effective IV iron (%)	Effective PO iron (%)
Erichsen et al. (2005) [16]	Norway	RCT	2	19	10	9	18-46	18-46	Both groups: 31.6%		15.8%	0.0%
Gisbert et al. (2009) [17]	Spain	Prospective study	24	100	22	78	Both groups: 40 (16)		Both groups: 35%		77.3%	88.5%
Howaldt et al. (2022) [18]	Multiple countries	RCT	12	250	125	125	40.4 (15.5)	40.0 (14.6)	38%	46%	56.0%	47.2%
Kulnigg et al. (2008) [19]	Multiple countries	RCT	12	196	136	60	NA	NA	NA	NA	75.9%	65.1%
Lindgren et al. (2009) [20]	Sweden	RCT	20	91	45	46	42.1 (15)	42.8 (16.5)	28.80%	32.60%	66.7%	47.8%
Reinisch et al. (2013) [21]	Multiple countries	RCT	8	327	219	108	36	35	37%	38%	65.3%	58.4%
Schröder et al. (2005) [22]	Germany	RCT	6	46	22	24	35	33	22.70%	29.20%	45.5%	37.5%

The seven included studies were published between 2005 and 2022 and comprised predominantly RCTs [16,18-22], with one prospective observational study [17]. Geographically, they spanned Europe and multinational settings: two studies were conducted in single European countries - Norway [16] and Sweden [20] - while others originated from Spain [17] and Germany [22]. The remaining studies were multinational collaborations involving centers across multiple countries [18,19,21]. Follow-up durations varied from short-term interventions of two weeks [16] to longer observation periods extending up to 24 weeks [17], with most studies following patients for 6-20 weeks.

A total of 1,029 participants were included across all studies, with individual sample sizes ranging from 19 [16] to 327 [21]. The allocation between IV and oral iron supplementation groups varied widely, from small-scale equal allocations, such as in Schröder et al. [22] (22 IV and 24 oral), to larger disparities, as seen in Gisbert et al. [17] (22 IV and 78 oral). The largest trial, conducted by Reinisch et al. [21], assigned 219 participants to IV therapy and 108 to oral therapy.

The mean age of participants ranged from the early 30s to the early forties when reported, with relatively similar age distributions between treatment arms [17,18,20-22]. Some studies provided ranges rather than means, such as Erichsen et al. [16], which included patients aged 18-46 years. Gender distribution varied, with male representation ranging from 22.7% in the IV arm of Schröder et al. [22] to 46% in the oral arm of Howaldt et al. [18]. Notably, some studies did not provide gender breakdowns [19], reflecting variation in reporting standards.

Across studies, the percentage of patients achieving an effective increase in hemoglobin levels tended to favor IV supplementation, though not universally. The highest IV efficacy was reported by Gisbert et al. [17] (88.5%), followed by Kulnigg et al. [19] (75.9%), while the lowest was seen in Erichsen et al. [16], where no patient in the IV group achieved the defined improvement. Oral supplementation was associated with comparatively lower efficacy in most trials, ranging from 37.5% [22] to 65.1% [19], though Gisbert et al. [17] reported a relatively high response rate in the oral group (77.3%). The observed variability in treatment effects underscores the heterogeneity in patient populations, baseline anemia severity, and intervention protocols across the included studies.

Quantitative Data Synthesis

**Figure 2:**
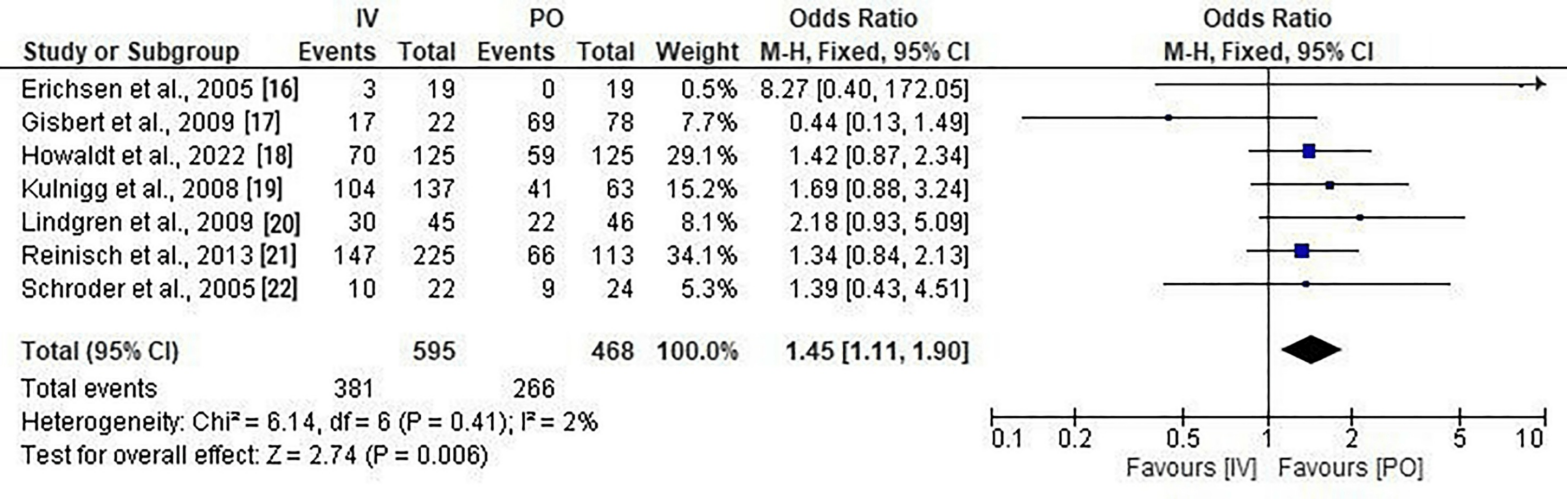
Forest plot for the comparison between IV and PO iron supplementation in inflammatory bowel disease patients, in terms of effective increase in hemoglobin levels

A meta-analysis of all seven studies demonstrated that IV iron supplementation was significantly more effective than oral iron in achieving a clinically relevant increase in hemoglobin levels among patients with IBD-associated anemia. The pooled OR was 1.45 (95% CI: 1.11 to 1.90; p = 0.006), favoring IV administration (Figure 2). This effect was consistent across most studies, with five out of seven showing ORs greater than 1. The largest contributions to the pooled effect came from Reinisch et al. [21] (34.1% weight) and Howaldt et al. [18] (29.1% weight).

Statistical heterogeneity was minimal, with a Chi-square value of 6.14 (df = 6, p = 0.41) and an I² statistic of 2%, indicating that the variation in results was largely due to chance rather than methodological or clinical differences among studies. This low heterogeneity supports the robustness of the pooled effect estimate.

Publication Bias

**Figure 3:**
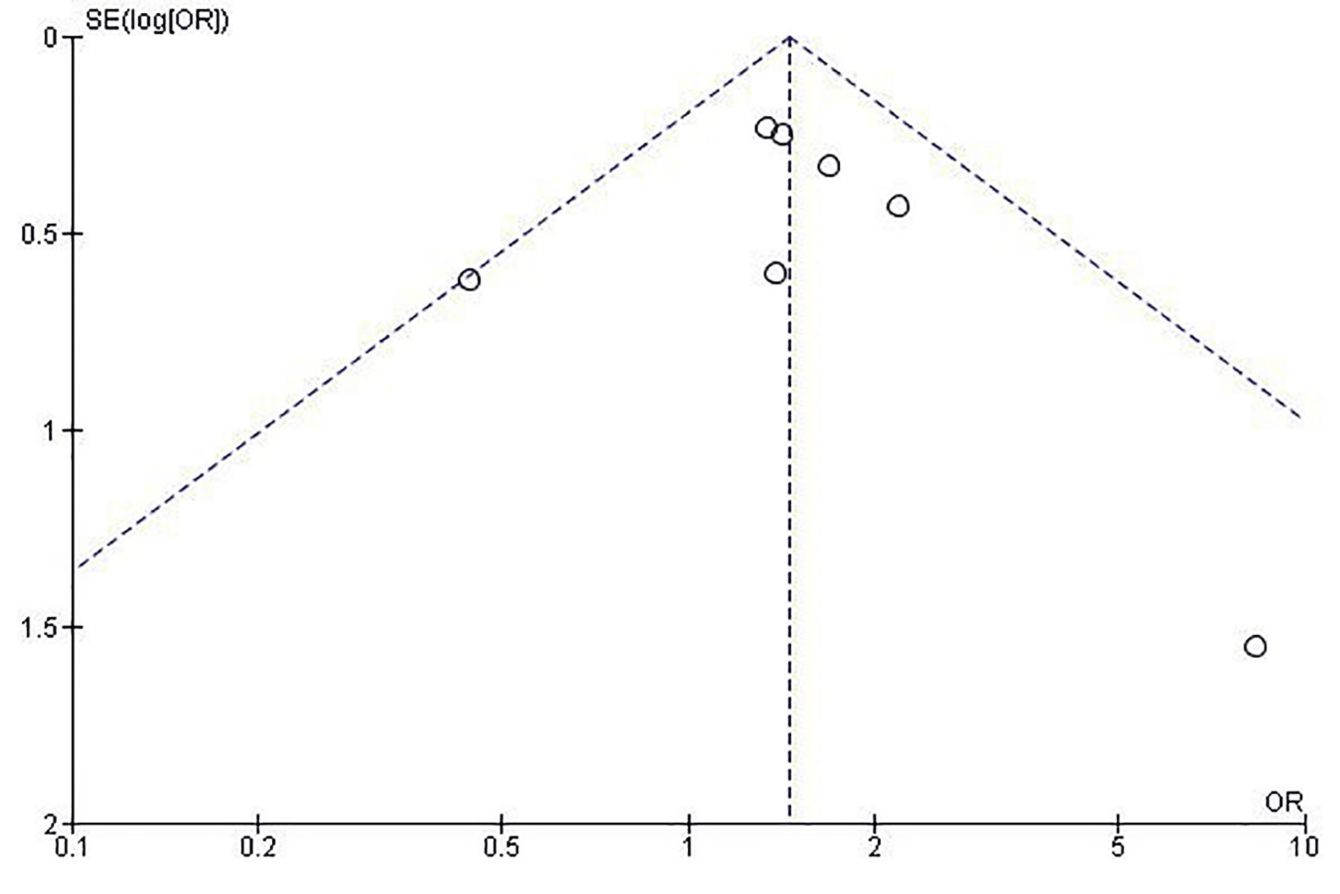
Funnel plot for the assessment of the publication bias

Visual inspection of the funnel plot revealed a symmetrical distribution of effect estimates (Figure 3), suggesting a low likelihood of publication bias. The symmetry indicates that both small and large studies were equally likely to be published, regardless of the direction or magnitude of their findings.

Risk of Bias Assessment

The risk of bias assessment revealed that the majority of the included studies demonstrated an overall low to moderate risk of bias. Most trials provided adequate details regarding the randomization process, with five studies clearly describing appropriate random sequence generation. Allocation concealment was adequately reported in four studies, while others did not provide sufficient methodological detail, leading to an unclear assessment in this domain. Blinding of participants and personnel was generally not feasible due to the distinct differences between oral and IV iron administration routes, resulting in a high risk of performance bias across all trials. However, the majority of studies reported complete outcome data and showed low risk for attrition bias. Selective reporting was assessed as low risk in most studies, although a few had unclear reporting of secondary outcomes. No significant concerns regarding other sources of bias were identified. Overall, the included studies were judged to be of acceptable methodological quality, providing reasonable confidence in the pooled estimates derived from the meta-analysis (Table 2).

**Table 2:** Risk of bias assessment for the included studies (n = 7) +, Low risk of bias; -, High risk of bias; ?, Unclear risk of bias

Study	Randomization process	Allocation concealment	Blinding of participants and personnel	Incomplete outcome data	Selective reporting	No other sources of bias
Erichsen et al. (2005) [16]	?	?	-	-	+	?
Gisbert et al. (2009) [17]	+	?	-	?	?	+
Howaldt et al. (2022) [18]	+	+	-	+	+	?
Kulnigg et al. (2008) [19]	+	+	-	+	+	+
Lindgren et al. (2009) [20]	+	?	-	?	+	+
Reinisch et al. (2013) [21]	+	+	-	+	?	+
Schröder et al. (2005) [22]	+	+	-	?	+	?

Discussion 

IDA is among the most prevalent extraintestinal complications of IBD, with prevalence estimates ranging from 36% to 76%, depending on disease activity, geographic region, and diagnostic criteria used [1,2]. Chronic intestinal inflammation, gastrointestinal blood loss, malabsorption, and the inhibitory effect of hepcidin on intestinal iron uptake contribute to anemia in this population [7]. Effective management of IDA in IBD is critical, as anemia significantly impacts quality of life, functional capacity, and disease prognosis [4,8]. Oral iron supplementation is widely used as a first-line treatment owing to its low cost and ease of administration; however, its gastrointestinal side effects, limited absorption during active inflammation, and risk of worsening oxidative stress in the mucosa have raised concerns [3,9]. IV iron formulations, on the other hand, bypass intestinal absorption and replenish iron stores more rapidly, but their higher cost, need for infusion facilities, and rare hypersensitivity reactions require careful consideration [11,23,24].

Over the last two decades, several RCTs and prospective studies have compared IV and oral iron supplementation in IBD-related anemia. However, the reported efficacy has been inconsistent across trials, partly due to differences in inclusion criteria, definitions of treatment response, and iron formulations used [17,18,20,21]. Against this background, our meta-analysis aimed to provide an updated, quantitative synthesis of the comparative efficacy of IV versus oral iron supplementation for anemic patients with IBD.

Our meta-analysis pooled data from seven studies [16-22], including 1,029 participants, to compare the efficacy of IV and oral iron supplementation in achieving a clinically significant increase in hemoglobin levels in IBD-associated anemia. The results demonstrated a statistically significant advantage for IV therapy, with a pooled OR of 1.45 (95% CI: 1.11-1.90; p = 0.006). This indicates that patients receiving IV iron were approximately 45% more likely to achieve a meaningful hemoglobin response compared with those receiving oral iron.

Heterogeneity was minimal (I² = 2%, p = 0.41), suggesting that the observed effect was consistent across different study designs, populations, and follow-up durations. The funnel plot showed a symmetrical distribution, suggesting a low likelihood of publication bias.

The superiority of IV iron supplementation observed in our meta-analysis aligns with the biological rationale that inflammation-mediated hepcidin upregulation in IBD reduces intestinal iron absorption, limiting the efficacy of oral iron [23,25]. The largest contributors to the pooled effect, Reinisch et al. [21] and Howaldt et al. [18], both reported higher response rates in the IV groups, with ORs of 1.34 and 1.42, respectively, consistent with the overall trend. Similarly, Kulnigg et al. [19] and Lindgren et al. [20] found greater hemoglobin responses with IV therapy, reporting effective response rates of 75.9% vs. 65.1% and 66.7% vs. 47.8%, respectively.

It is worth noting that Gisbert et al. [17] observed relatively high efficacy in the oral group (77.3%), which reduced the magnitude of difference between groups (OR 0.44). This may be attributable to the longer follow-up period of 24 weeks, allowing for adequate iron repletion with oral therapy in selected patients with lower baseline inflammation. Erichsen et al. [16], conversely, reported no effective response in the IV group over a short, two-week follow-up, highlighting that very short durations may underestimate IV therapy benefits [26].

Although an OR of 1.45 represents a moderate effect, its clinical significance should not be underestimated. In the context of IBD, where ongoing blood loss and malabsorption are common, achieving even a modestly higher likelihood of hemoglobin normalization can translate into improved patient-reported outcomes, reduced fatigue, and better tolerance of immunosuppressive therapies [27,28]. Furthermore, the minimal heterogeneity observed in our analysis suggests that this benefit is consistent across various patient subgroups, including those from different geographic regions and disease severities.

The biological plausibility of our findings is supported by the pathophysiology of iron metabolism in IBD. During active inflammation, elevated interleukin-6 stimulates hepatic production of hepcidin, which degrades ferroportin, the only known iron exporter from enterocytes, thereby reducing dietary iron absorption [3,11,28]. Oral iron must pass through this absorption pathway, whereas IV iron bypasses it entirely, delivering iron directly to the reticuloendothelial system, and allowing rapid erythropoiesis. This mechanistic advantage likely underpins the superior efficacy observed with IV therapy in our pooled analysis [24,27].

Current ECCO guidelines recommend IV iron as first-line therapy for patients with moderate-to-severe anemia (hemoglobin <10 g/dL), active disease, or intolerance to oral formulations [2]. Our findings support this recommendation by demonstrating superior efficacy with IV iron, even in mixed populations that include patients with quiescent disease. However, the fact that oral iron achieved substantial response rates in some trials (e.g., Gisbert et al. [17]) suggests that it remains a reasonable option for selected patients with mild anemia, inactive disease, and good tolerance [2,26].

The main strengths of this review include the comprehensive search across six major databases, the inclusion of only studies directly comparing IV and oral iron in IBD populations, and rigorous quantitative synthesis, with minimal statistical heterogeneity. The large pooled sample size enhances the precision of our estimates, and the absence of substantial publication bias increases the credibility of our findings.

For clinicians, these findings underscore the importance of tailoring iron supplementation strategies to individual patient profiles. While IV iron appears to offer superior efficacy overall, the choice of therapy should also consider cost, resource availability, patient preference, and safety profiles. IV formulations may be prioritized for patients with active inflammation, intolerance to oral iron, or those requiring rapid correction of anemia. Conversely, oral iron remains a viable first-line option for stable patients with mild anemia, and no significant gastrointestinal intolerance.

## Conclusions

In summary, this meta-analysis of seven studies involving 1,029 patients with IBD-associated anemia demonstrates that IV iron supplementation is significantly more effective than oral iron in achieving a clinically meaningful increase in hemoglobin levels (OR 1.45, 95% CI: 1.11-1.90; p = 0.006), with minimal heterogeneity. These findings are consistent with current pathophysiological understanding and clinical guidelines, reinforcing the role of IV iron as the preferred option for patients with active disease, intolerance to oral formulations, or those requiring rapid correction of anemia. Oral iron remains a reasonable alternative for selected patients with mild, stable disease.
